# Pandora's Box

**DOI:** 10.1192/bji.2021.44

**Published:** 2021-11

## A coffee a day keeps the doctor away

Good day to all readers, and feel free to enjoy your coffee as you read this. If you, like Pandora, are a coffee afficionado, read on and feel good, and if you are not, you may consider adding coffee to your healthy lifestyle. Pandora praised the health benefits of coffee in previous issues when she presented evidence that it protects your liver, even in heavy alcohol drinkers (can't do wonders though!) and helps you live longer. There is now strong evidence that it can lower your risk of stroke and cardiovascular disease.

The lead researcher from the Heart and Vascular Centre at Semmelweis University in Budapest, Hungary, made these claims, based on the largest study to systematically assess the cardiovascular effects of regular coffee consumption. The study participants, close to half a million, males and females with an average age of 56, were from the UK Biobank and had no signs of heart disease at the time of recruitment. They were divided into three groups according to their usual coffee intake: (a) none = no regular coffee intake (22.1%); (b) light to moderate intake = 0.5 to three cups a day (58.4%); and (c) high intake = more than three cups a day (19.5%). Using a multivariable model analysis over a median follow-up of 11 years, adjusted for relevant factors including age, sex, weight, height, smoking status, physical activity, high blood pressure, diabetes, cholesterol level, socioeconomic status, and usual intake of alcohol, meat, tea, fruit and vegetables, the researchers found that light to moderate coffee drinkers had a 12% lower risk of all-cause death compared with non-drinkers, a 17% lower risk of death from cardiovascular disease and a 21% lower risk of a stroke incident.

Searching for a possible mechanism for these positive effects, they assessed cardiac status in a subgroup of 30 650 subjects who underwent cardiac magnetic resonance imaging. They found that daily coffee drinkers had healthier and better functioning hearts, a finding they considered consistent with reversing the detrimental effects of ageing on the heart.

Pandora will be looking out for the full study when published and update Pandora's Box accordingly. It would be interesting to know what the outcomes were in the third group, that of the heavy coffee drinkers. In the meantime, best to stick to moderate consumption!

Simon J. Association of daily coffee consumption with cardiovascular health – results from the UK Biobank. ESC Congress 2021.

## Muscle burn fires the brain

A lot has been said about the beneficial effects of exercise on our brains and that it helps prevent or delay Alzheimer's dementia, a nemesis in our ageing years. We know that aerobic exercise enhances blood flow to the brain and increases grey and white matter volume, hence improving cognition. Being able to measure the effects of exercise on biomarkers associated with increased risk of Alzheimer's dementia could enable measurement of the effects of exercise on the brain and hopefully in the long run help in the prevention, monitoring and treatment of this devastating condition. A very recent study addresses this issue by examining specifically the role of such systemic biomarkers.

The investigators used blood samples from 23 asymptomatic males and females with a mean age of 65, all with familial and genetic risk for Alzheimer's dementia, taken before and after 26 weeks of supervised treadmill training. They measured systemic biomarkers thought to be involved in learning and memory via their effects on the brain (the hippocampus in particular), on neurogenesis and neuroplasticity, i.e. myokine cathepsin B (CTSB), brain-derived neurotrophin and klotho, as well as metabolomics.

They found an increase in levels of CTSB and changes in lipid metabolites implicated in dementia, as well as in the gut microbiome, indicating a positive association between exercise and cognition in support of the beneficial effects of exercise on brain function.

Get off that couch and start exercising!

Gaitán JM, Moon HY, Stremlau M, Dubal DB, Cook DB, Okonkwo OC,  Effects of aerobic exercise training on systemic biomarkers and cognition in late middle-aged adults at risk for Alzheimer's disease. Front Endocrinol 2021; 12: 660181.10.3389/fendo.2021.660181PMC817316634093436

## Do we really care?

We all like to think we are caring and compassionate people. Compassion is so much needed at present, with the increasing and vast economic differences between and within countries in the world, multiple conflict areas, the climatic changes and their consequences, and the yet to be controlled Covid pandemic.

Researchers in California claim a difference between compassion and empathy, with compassion meaning having feelings of caring or sympathy for another person and empathy thought to involve taking on another person's suffering and experiences as if they were one's own (by the way, the word empathy has a very different meaning in its original ancient and modern Greek language to the one given in English, but this is probably of no interest to the reader, so let it be!).

They examined the question via a series of studies involving a variable number of subjects from 62 to 215. The participants were presented with cards showing images of persons suffering and given three different card decks to choose from: (a) asking them to feel compassion for the person in the card; (b) asking them to feel empathy; and (c) asking them to remain objective and simply describe the person.

In the study that compared the two alone, they found that people were more likely to feel empathy than compassion, although overall the studies found the preference was for the participants remaining objective. All studies revealed that the participants opted to avoid compassion when given the opportunity. The reason given was that compassion was more cognitively taxing than empathy and objective detachment. However, the subjects were more likely to feel compassion for close (family, friends) than distant others, as they considered this less cognitively taxing. They also found that when the pleas for help they were presented with were richer in context and more immersive, the participants preferred to escape feeling compassion.

The authors conclude that ‘finding ways to better manage the mental challenges of compassion may provide a more rewarding route to generating prosocial motivation, especially in this particularly troubling time’. What does this say about human nature? Do we really lack compassion so much that we need to work on it? Have we become ‘immune’ to compassion in our modern world, saturated by being constantly bombarded by the media with images of suffering? What happened to our humanity?

Scheffer JA, Cameron CD, Inzlicht M. Caring is costly: people avoid the cognitive work of compassion. J Exp Psychol Gen [Epub ahead of print] 19 Aug 2021. Available from: 10.1037/xge0001073.34410802

## ‘Who pays the piper’

We are all aware of possible bias in studies funded by private agencies such as the pharmaceutical industry. But should we have blind faith in research supported by government-funded national agencies (such as the National Institute of Health in the USA and the National Health Research Council in Australia), other governmental agencies such as local councils, public health and safety departments, and ministerial departments, state-funded projects by industry, and philanthropic organisations?

A recent survey among public health researchers suggests otherwise. The authors claim that such agencies that are responsible for giving policy advice or implementing intervention programmes have a stake in the findings, and this may lead them to apply pressure on researchers, influencing their impartiality. The aim of the survey was to explore the prevalence of efforts by funders to suppress trial findings on health behaviour interventions.

They invited the lead or corresponding authors of studies published between 2007 and 2017 that had been included in a Cochrane review to a computer-assisted telephone interview or online survey. These were studies on interventions to improve nutrition, physical activity, sexual health, smoking and substance use. The participants were asked a number of questions on possible suppression of aspects of reporting.

A total of 104 authors responded (50% of those approached) of 208 trials from North America – USA and Canada (34%), Europe (33%), Oceania (17%) and elsewhere in the world (16%). The participants were asked seven questions on their experience of pressure to suppress when disseminating the trial results, with the options being ‘not at all’, ‘a little’ and ‘substantially’.

The authors' responses showed a variety of ways the funders attempted to interfere in the publication of the results of the intervention trials. Two-thirds of the studies were conducted in North America or Europe/UK. The Economist Intelligence Unit (EIU), was used as the democracy index to categorise countries as full democracy (scores = 8.01 to 10) or not full democracy (scores = 0 to 8). The EIU is a recognised and complex measure of a country's democracy level and is based on five categories of 60 indicators, which are scored to provide a maximum total out of ten. The majority of studies were from full democracy countries (61%).

One-fifth of the respondents reported that on at least one occasion they felt under pressure by funders to delay, alter or not publish findings. The most commonly reported forms of suppression were the funder expressing reluctance to publish because they considered the results unfavourable (9%), requesting conclusions to be altered (6%) or reporting of findings to be delayed (5%), attempts to discredit members of the research team (4%), requesting unfavourable findings not to be published (3%), discouraging presentation of results to particular groups (3%), and demanding changes to methods or analysis (2%).

McCrabb S, Mooney K, Wolfenden L, Gonzalez S, Ditton E, Yoong S, . “He who pays the piper calls the tune”: researcher experiences or fund suppression of health behaviour intervention trial findings. PLoS One 2021; 16(8): e0255704.3440710410.1371/journal.pone.0255704PMC8372890

## Delaying retirement is good for you

As global economy was going through a major crisis, governments across the world found the opportunity to increase the retirement age to 67. Those of you nearing retirement and feeling disgruntled by this, cheer up! It may be just good for you, at least so say researchers from the Max Plank Institute for Demographic Research in Rostock, Germany.

They used data on over 20 000 people from the Health and Retirement Study of the US, between 1996 and 2014, to estimate the effects of retirement. In an attempt to disentangle the effects of postponing retirement on later-life cognition from the effects of other life-course factors, they examined the effects of gender, education and occupation and also whether retirement affects cognitive function via depressive symptoms or comorbidities.

The results showed that postponing retirement does indeed protect against cognitive decline. They also found that those with the highest education had the greatest mitigation of cognitive decline.

Let's hope there was no pressure from the funders to present the data in this way!

Hale JM, Bijlsman MJ, Lorenti A. Does postponing retirement affect cognitive function? A counterfactual experiment to disentangle life course risk factors. SSM Popul Health 2021; 15: 100855.3425837510.1016/j.ssmph.2021.100855PMC8255239

